# LINC02678 as a Novel Prognostic Marker Promotes Aggressive Non-small-cell Lung Cancer

**DOI:** 10.3389/fcell.2021.686975

**Published:** 2021-05-28

**Authors:** Dexin Jia, Ying Xing, Yuning Zhan, Mengru Cao, Fanglin Tian, Weina Fan, Jian Huang, Yimeng Cui, Ruixue Gu, Yaowen Cui, Yuechao Liu, Shuai Zhang, Li Cai, Xiaomei Li

**Affiliations:** ^1^The Fourth Department of Medical Oncology, Harbin Medical University Cancer Hospital, Harbin, China; ^2^Department of Pathology, Harbin Medical University Cancer Hospital, Harbin, China

**Keywords:** LINC02678, enhancer of zeste homolog 2, non-small-cell lung cancer, proliferation, epithelial-mesenchymal transition

## Abstract

Non-small-cell lung carcinoma (NSCLC) is considered to be a fatal disease and characterized by a poor prognosis. Long non-coding RNAs (lncRNAs) have been reported to act as biomarkers and therapeutic targets in solid tumors. However, the expression of lncRNAs and their clinical relevance in NSCLC remain undetermined. The gene expression data profiled in The Cancer Genome Atlas and Gene Expression Omnibus (GSE81089) were employed to screen differentially expressed lncRNAs in NSCLC. LINC02678 was found to be upregulated in NSCLC and exhibited hypomethylation of the promoter region in NSCLC tissues. LINC02678 (also called RP11-336A10.5) was associated with poorer overall survival and relapse-free survival in NSCLC patients. *In vitro* models of gain- and loss-of-function demonstrated that LINC02678 promotes NSCLC progression by promoting NSCLC cell proliferation and cell cycle progression, as well as inducing NSCLC cell migration, invasion and epithelial-mesenchymal transition. LINC02678 was primarily located in the nucleus and could bind with the enhancer of zeste homolog 2 (EZH2). Moreover, we found that LINC02678 knockdown impaired the occupancy capacity of EZH2 and trimethylation of lysine 27 on histone 3 (H3K27me3) at the promoter region of cyclin dependent kinase inhibitor 1B (CDKN1B) and E-cadherin, as confirmed by ChIP-qPCR. A mouse transplantation model further demonstrated that LINC02678 could promote the tumorigenic and metastatic capacities of NSCLC cells. We identified LINC02678 as a tumor promoter in NSCLC, which enhanced the growth and metastasis of NSCLC cells by binding with EZH2, indicating that LINC02678 may serve as a potential biomarker for cancer diagnosis and treatment.

## Introduction

Lung cancer is the most common malignancy and the leading cause of cancer-related deaths worldwide. It causes more than 1.76 million associated deaths, 18.4% of all cancer deaths (Bray et al., 2018). Non-small cell lung cancer (NSCLC) is the main histological type of lung cancer (over 85%). The 5-year survival rate of NSCLC patients is 19% (Siegel et al., 2020). Once the cancer has spread, it is often shown a limited survival (Zheng et al., 2020). Therefore, it is urgent to seek effective biomarkers for the early diagnosis and individualized treatment of NSCLC.

During the last decade, whole genome sequencing has identified a large number of long non-coding RNAs (lncRNAs) that are involved in multiple biological processes such as cancer development, embryonic stem cell pluripotency and cell cycle regulation through transcriptional interference, induction of intracellular chromatin remodeling and histone modifications within cells (Rinn and Chang, 2012). LncRNAs have been reported to act as oncogenes or cancer-suppressors across solid tumors (Bhan et al., 2017). For instance, HOX transcript antisense intergenic RNA is a lncRNA essential for the growth of breast cancer cells and is closely associated with the migration and invasion of breast cancer cells (Gupta et al., 2010). Urothelial cancer associated 1 is a promising urine biomarker for non-invasive diagnosis of bladder carcinoma (Huang et al., 2016). Metastasis associated lung adenocarcinoma transcript 1 (MALAT1) is upregulated in several cancers, including lung, bladder, breast, prostate and ovarian cancers, and serves as a potential biomarker and therapeutic target (Arun et al., 2016). Furthermore, recent studies have shown that lncRNAs can interact with enhancer regions, leading to increased activity of neighboring genes (Ørom and Shiekhattar, 2011). The focus on these lncRNAs may inspire developing new cancer therapeutic targets.

In this study, we aimed to identify lncRNAs that are upregulated in NSCLC tissues and affecting the prognosis of NSCLC patients, based on The Cancer Genome Atlas (TCGA) and Gene Expression Omnibus (GEO) database. We observed that high level of LINC02678 (also called RP11-336A10.5) showed an association with poor survival of NSCLC patients. Based on *in vitro* experiments, we investigated and characterized the binding potential between LINC02678 and the enhancer zeste homolog 2 (EZH2) and its influence on the proliferation and migration ability of NSCLC cell lines. The effects of LINC02678 on tumorigenesis, cancer cell growth and metastasis were further investigated based on animal experiments. Our findings point to a role for LINC02678 as an oncogene in NSCLC and provide a theoretical basis for identifying and developing new diagnostic and therapeutic biomarkers.

## Materials and Methods

### Bioinformatic Analysis

All bioinformatics analyses were completed using R v 3.6.3. One cohort was consisted of 199 human NSCLC samples and 19 normal samples from GEO.^1^ Other cohorts including transcriptome data of human LUAD, LUSC and normal samples were obtained from the TCGA^2^ database (Tomczak et al., 2015; Wang et al., 2016). According to the following criteria, differentially upregulated lncRNAs between NSCLC tissue samples and normal lung tissue samples based on TCGA and GEO datasets were selected: *p*-value <0.05 and fold change >1.5. In order to explore the impact of these up-regulated lncRNAs on survival of NSCLC patients, the Kaplan-Meier method was used to perform a survival analysis of OS and RFS using the “survival” package, and the log-rank test was applied to test the survival data. LncRNAs that had prognostic significance on both OS and RFS in LUAD and LUSC patients (*p* < 0.05) were retained, and all others were excluded. DNA methylation data for NSCLC tissues and normal tissues were retrieved from TCGA’s Illumina Infinium HumanMethylation450 Beadchip dataset, and the methylation levels of probes for LINC02678 in LUAD and LUSC was calculated as beta value and differential methylation was assessed and visualized by R.

### Tumor Xenograft Implantation

For *in vivo* experiments, A549 cell lines stably transfected with negative control (NC) and shLINC02678-1 (shLINC02678) were first established. Four-to-five-week-old BALB/c mice were purchased from Beijing Vital River Laboratory Animal Technology Co., Ltd. and raised at the Animal Center of the Second Affiliated Hospital of Harbin Medical University. For proliferation analysis (*n* = 5 mice/group), the cells were implanted into the right flank of the mice, and bioluminescence images were obtained at day 28 post-implantation. Tumor volumes were measured every three days. For metastasis analysis (*n* = 6 mice/group), the cells were injected into the tail vein of the mice. And bioluminescence images were obtained at day 60 post-implantation.

### Cell Culture and Collection of NSCLC Samples

Human bronchial normal epithelial cell lines (HBE) and NSCLC cell lines (PC9, 95D, H292, A549, HCC827, H1299, and H1650) were cultured in RPMI-1640 or DMEM medium supplemented with 10% fetal bovine serum and maintained in an incubator set to 37°C and 5% CO_2_ conditions. Fresh frozen tumor and adjacent normal tissue samples were excised from eight NSCLC patients receiving pneumonectomy at the Affiliated Cancer Hospital of Harbin Medical University. Ethical clearance and approval were obtained from the Ethics Review Committee of Harbin Medical University.

### Cell Transfection and Quantitative Real-Time PCR (RT-qPCR)

Cells were transfected with lentiviruses with overexpressed or knockdown sequences of LINC02678 purchased from GeneChem (Shanghai, China), and screened with puromycin. Knockdown of EZH2 was conducted by using small interfering RNA (siRNA; RiboBio, China). E.Z.N.A. Total RNA Kit I (R6834-01, Omega Bio-Tek, United States) was used to extract RNA from the cells and tissues. The expression level of RNA was determined by RT-qPCR with glyceraldehyde 3-phosphate dehydrogenase (GAPDH) gene as the control. Primer sequences are presented in the Supplementary Table 4.

### Cell Proliferation and Viability Assays

CCK-8 assays were conducted using CCK-8 kit (Dojindo, Japan) with absorbance detection at 450 nm. EdU incorporation assay was performed using Cell-Light ^TM^ EdU Apollo567 *In Vitro* Kit (Catalogue Number C10310–1, RiboBio, China) according to the manufacturer’s instructions, and images were captured by fluorescence microscopy. For colony formation assays, 1,000 cells were seeded into 6-well plates and cultured in complete medium for a fortnight. Colonies were then fixed in methanol and stained with 0.5% crystal violet.

### Cell Cycle Analysis

Cells were treated with a Cell Cycle Staining Kit (70-CCS012, MultiSciences, China); the proportion of cells in different cell cycles was determined by the BD FACSCalibur flow cytometer.

### Tumor Cell Migration and Invasion Assays

The wound-healing assay and Transwell assay for tumor cell migration and invasion were performed as previously described (Cui et al., 2020).

### Western Blotting

Total protein from tissues or cells was extracted using radio-immunoprecipitation assay (RIPA) and was quantified by using the BCA Protein Assay Kit. Protein samples (30 μg) were separated by polyacrylamide electrophoresis, transferred onto polyvinylidene fluoride (PVDF) membranes and incubated with specific antibodies (listed in the Supplementary Table 4).

### Subcellular Fractionation

The Cytoplasmic and Nuclear RNA Purification Kit (21000, Norgen Biotek, Canada) was used for subcellular fractionation of 95D and A549 cells to determine the subcellular localization of LINC02678. RT-qPCR was performed to quantify LINC02678 expression, and GAPDH and U1 genes were used as the reference for cytoplasmic and nuclear RNA, respectively.

### Fluorescence *in situ* Hybridization

Fluorescence *In Situ* Hybridization Kit (10910, RiboBio Co., Ltd, China) was used to confirm the location of LINC02678 in the cells. According to the manufacturer’s instructions, cell suspensions were incubated on coverslips, followed by adding anti-LINC02678 and anti-U6 oligonucleotide probes into the suspensions for hybridization, and then DAPI staining was conducted. Finally, images were captured under a confocal laser scanning microscope (FV1200, Olympus, Japan).

### RNA Immunoprecipitation (RIP) Assay

The RNA-Binding Protein Immunoprecipitation Kit (YXZX-006, Wuhan GeneCreate Biological Engineering Co., Ltd., China) and the anti-EZH2 antibody were used to detect the binding potential between EZH2 and LINC02678 according to the manufacturer’s instructions.

### Chromatin Immunoprecipitation (ChIP)-PCR

Cells were collected and cleaved by sonication to generate DNA fragments. Antibodies and the SampleChIP^®^ Enzymatic Chromatin IP Kit (9003S, CST, United States) were used for the ChIP experiments. The purified DNA was analyzed for enrichment efficiency by PCR.

### Statistical Analysis

Data in this study were processed with GraphPad Prism 8.0.2. The means of normally distributed continuous data between two groups were analyzed by Student’s *t*-test. The differences in categorical variables among different groups were analyzed with χ^2^ tests. All data were determined in triplicate and are representative of at least two separate experiments. All data are shown as mean ± SEM. Differences were considered significant if *p* < 0.05. The survival distribution of the samples was assessed by the Kaplan-Meier method and analyzed using the log-rank test.

## Results

### Identification of LINC02678 as a Potential Oncogenic Factor in NSCLC

**FIGURE 1:**
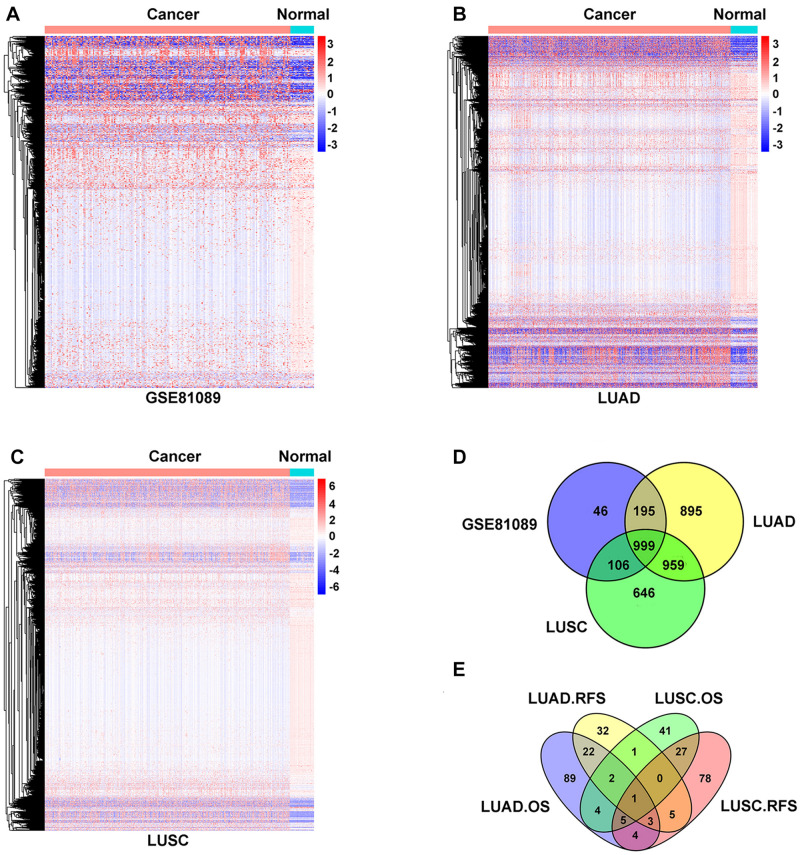
Identification of differentially expressed oncogenic lncRNAs in NSCLC. **(A–C)** Hierarchical clustering heat map of the upregulated lncRNAs of NSCLC samples compared with normal lung samples from TCGA (LUAD and LUSC) and GSE81089, with absolute fold changes >1.5 and *p*-value <0.05 as significant. **(D)** Venn diagram of the intersection of lncRNAs upregulated in TCGA (LUAD and LUSC) and GSE81089 databases. **(E)** Venn diagram of the intersection of OS and RFS in survival analysis of LUAD and LUSC patients expressing LINC02678 based on the TCGA database.

In this study, we aimed to identify lncRNAs that play an oncogenic role in NSCLC. Compared to normal tissue, 1,346 lncRNAs were upregulated in GSE81089 dataset (Figure 1A and Supplementary Table 1), 3,048 lncRNAs were upregulated in TCGA-LUAD dataset (Figure 1B and Supplementary Table 2), and 2,710 lncRNAs were upregulated in TCGA-LUSC dataset (Figure 1C and Supplementary Table 3), of which 999 lncRNAs were shared among all these three datasets (Figure 1D). Among these 999 lncRNAs, LINC02678 appeared to be a unique prognostic factor for both overall survival (OS) and relapse-free survival (RFS) in lung adenocarcinoma (LUAD) and lung squamous cell carcinoma (LUSC) patients by Kaplan-Meier analyses (Figure 1E).

**FIGURE 2:**
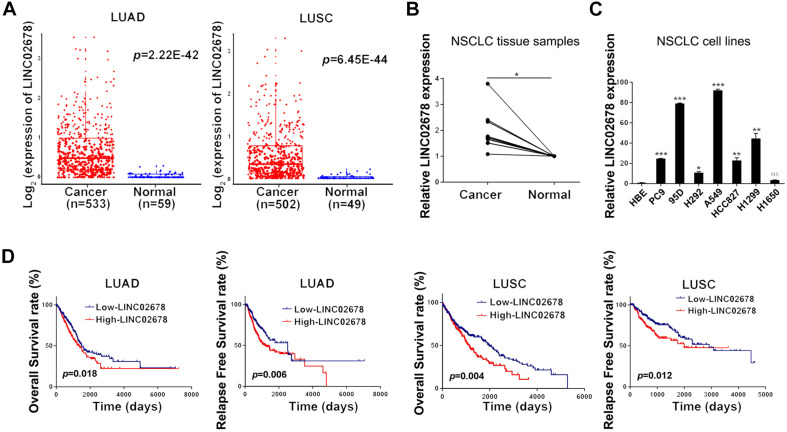
LINC02678 acts as a differentially expressed tumor promotor lncRNA. **(A)** The expression level of LINC02678 in human LUAD, LUSC tissues and normal lung samples. **(B)** LINC02678 expression in the paired lung tissues measured by RT-qPCR. **(C)** LINC02678 expression in normal bronchial epithelial cell line HBE and NSCLC cell lines measured by RT-qPCR. **(D)** Kaplan-Meier analysis of OS and RFS of LINC02678 in LUAD and LUSC. (*p < 0.05; **p < 0.01; ***p < 0.001. Data were obtained from three independent experiments).

Promoter hypomethylation can mediate oncogene activation. We found that LINC02678 was highly expressed (Figure 2A) and exhibited promoter hypomethylation (Supplementary Figure 1A) in human NSCLC samples in TCGA datasets. We also found that LINC02678 had a higher expression in NSCLC tissues than in paired non-tumor lung tissues (Figure 2B); NSCLC cell lines had a higher transcript level of LINC02678 compared to the normal bronchial epithelial cell line HBE (Figure 2C). Survival distribution depicted by Kaplan-Meier method showed that patients with high LINC02678 expression had shorter OS and RFS than those with low LINC02678 expression (Figure 2D).

### LINC02678 Promotes Proliferation and the G1/S Transition

To further explore the role of LINC02678 in NSCLC, 95D and A549 cells were used for loss-of-function assays while H292 and H1650 cells were used for gain-of-function assays considering the relative levels of LINC02678 in different NSCLC cell lines (Figure 2C).

**FIGURE 3:**
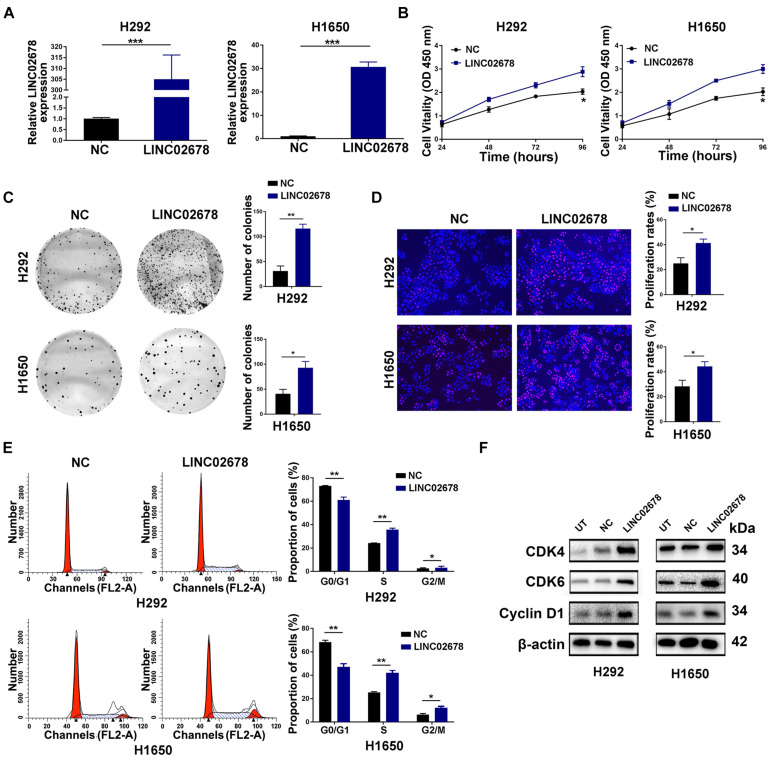
LINC02678 promotes proliferation and G1/S transition of NSCLC cells. **(A)** RT-qPCR analysis showed the expression of LINC02678 in H292 and H1650 cells transfected with negative control (NC) or overexpressed LINC02678 (LINC02678) lentivirus. **(B)** CCK-8 analysis showed the proliferative ability of H292 and H1650 cells in NC and LINC02678. **(C,D)** Colony formation and EdU analysis showed the proliferative ability of H292 and H1650 cells in NC and LINC02678 groups. **(E)** Flow cytometry analysis showed the proportion of H292 and H1650 cells in G0/G1, S and G2/M phases in NC and LINC02678 groups. **(F)** Western blotting analysis showed the expression of key cell cycle-related protein (CDK4, CDK6 and CyclinD1) in NC and LINC02678 groups. (*p < 0.05; **p < 0.01; ***p < 0.001. Data were obtained from three independent experiments).

Using lentiviral transfection, the cell line with over-expressed LINC02678 was efficiently established (Figure 3A). Sustainable cell proliferation is a hallmark of cancer progression (Hanahan and Weinberg, 2011). As demonstrated by the results of CCK-8 as well as the long-term colony formation and EdU incorporation assays, overexpression of LINC02678 significantly enhanced the proliferative capacity and viability of NSCLC cells compared to NC (Figures 3B–D). Furthermore, the proliferation of cancer cells was controlled by the cell cycle (Pack et al., 2019). Overexpression of LINC02678 significantly increased the proportion of S-phase cells compared to controls (Figure 3E), as well as upregulated the expression of the key cell cycle-related proteins cyclin-dependent kinase 4 (CDK4), cyclin-dependent kinase 6 (CDK6) and Cyclin D1 in H292/H1650 cells (Figure 3F). On the contrary, knockdown of LINC02678 slowed cell proliferation and increased the proportion of G0/G1-phase cells (Supplementary Figures 2A–F). These results suggested that LINC02678 could accelerate cell cycle progression, resulting in the uncontrolled proliferation of NSCLC cells.

### LINC02678 Promotes Migration, Invasion and Induces Epithelial-Mesenchymal Transition (EMT) in NSCLC Cells

**FIGURE 4:**
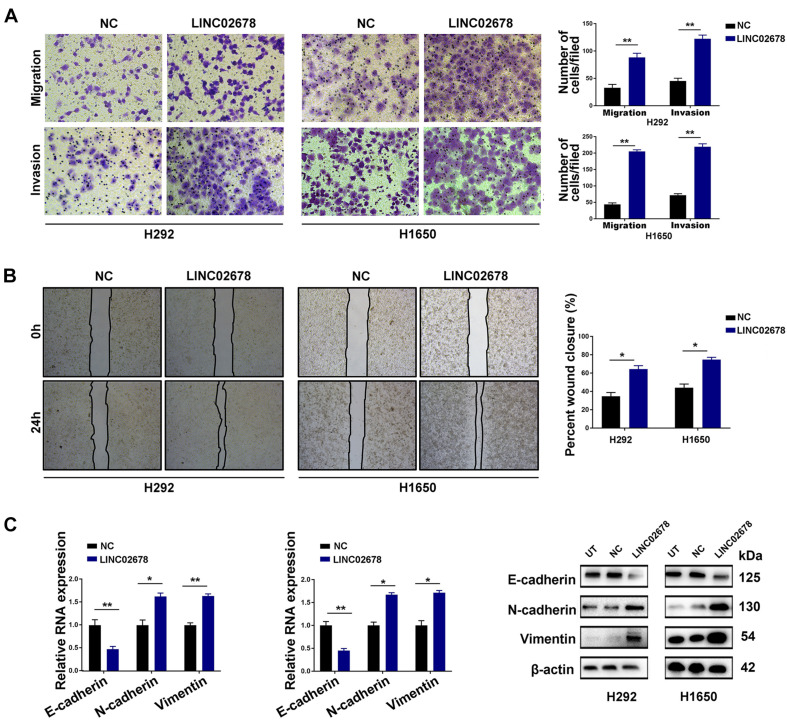
Up-regulated LINC02678 expression promotes migration, invasion and EMT of NSCLC cells. **(A,B)** The effects of up-regulated LINC02678 expression on the migration and invasion of H292 and H1650 cells were evaluated by wound healing experiment and Transwell assay. **(C)** Evaluation of EMT-related markers was performed by RT-qPCR and western blotting. (*p < 0.05; **p < 0.01. Data were obtained from three independent experiments).

Metastasis has been largely attributed to tumor recurrence and mortality of NSCLC patients (Wood et al., 2014). The invasive and migratory capacity of tumor cells reflects the metastatic potential of cancer (Hanahan and Weinberg, 2011). Through wound healing and Transwell assays, we explored the effect of LINC02678 on the migratory and invasive capacity of NSCLC cells. As expected, overexpression of LINC02678 increased the migration and invasion of H292 and H1650 cells compared to the NC (Figures 4A,B), while LINC02678 knockdown exhibited the opposite results (Supplementary Figures 3A,B). Besides, EMT is one of the essential steps for initiating cancer metastasis, and E-cadherin, N-cadherin, and vimentin are considered as important EMT-related proteins (Serrano-Gomez et al., 2016). Overexpression of LINC02678 induced a decrease in the epithelial marker E-cadherin and an increase in the mesenchymal-associated proteins N-cadherin and vimentin, both at the transcriptional or translational level (Figure 4C), whereas knockdown of LINC02678 induced E-cadherin overexpression and decreased the expressions of N-cadherin and vimentin (Supplementary Figure 3C).

### LINC02678 Binds With EZH2 and Modulates the Expression of CDKN1B and E-Cadherin

**FIGURE 5:**
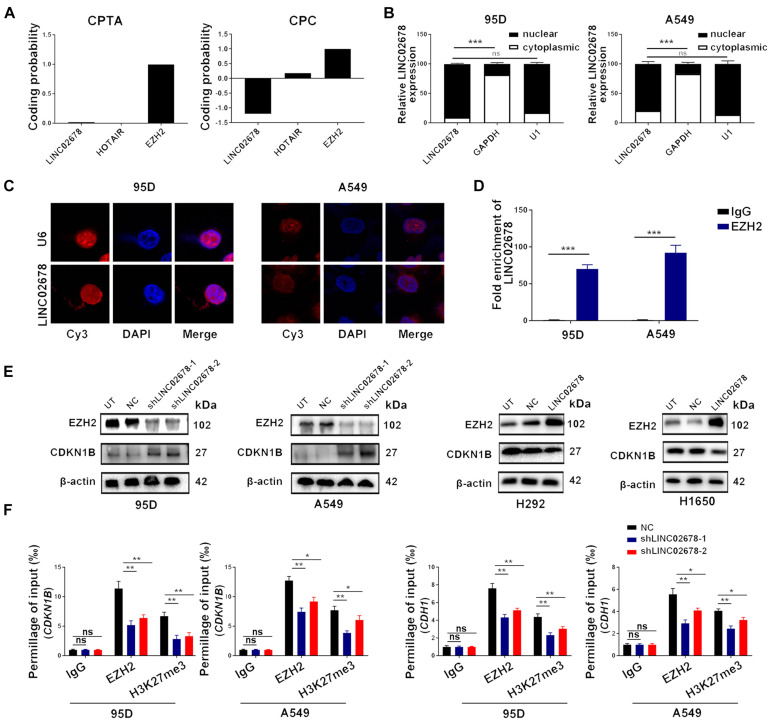
LINC02678 is a nuclear-localized transcript and knockdown of LINC02678 represses EZH2 expression and activity. **(A)** Protein-coding potential of LINC02678 analyzed through the CPAT database and the CPC database. **(B)** RT-qPCR analysis of the relative RNA expression levels after nuclear and cytoplasmic RNA separation. GAPDH was used as a cytoplasmic marker and U1 was used as a nuclear marker. **(C)** Representative FISH images of the subcellular location of LINC02678 in 95D and A549 cells (red). Nuclei were stained with DAPI (blue). U6 was used as a nuclear marker. **(D)** RIP and RT-qPCR analysis of endogenous EZH2 bound with LINC02678 in 95D and A549 cells. IgG was used as the control group. **(E)** Western blotting shows the protein expression of EZH2 and CDKN1B after knockdown or overexpression of LINC02678. **(F)** CHIP-qPCR analysis of EZH2 and H3K27me3 occupancy on the E-Cadherin (CDH1) and CDKN1B promoter region in A549 and 95D cells after transfected with NC, shLINCC02678-1 or shLINC02678-2. (*p < 0.05; **p < 0.01; ***p < 0.001. Data were obtained from three independent experiments).

Consistent with other members of long non-coding RNAs, LINC02678 possesses no protein-coding capacity, as demonstrated by the Coding Potential Assessment Tool (CPAT)^3^ and the Coding Potential Calculator (CPC)^4^ (Figure 5A). To clarify the role of LINC02678 in NSCLC cells, subcellular localization assays of LINC02678 were performed through nuclear and cytoplasmic RNA fractionation analysis, RNA fluorescence *in situ* hybridization was conducted, which confirmed that LINC02678 was predominantly localized in the nucleus (Figures 5B,C).

Growing numbers of evidence has demonstrated that nuclear lncRNAs can regulate gene expression by binding with polycomb repressor complex 2 (PRC2), which is composed of EZH2 (Su et al., 2018). The binding potential between LINC02678 and EZH2 in 95D and A549 cells was confirmed by RIP experiments (Figure 5D). Furthermore, *in vitro* experiments showed that LINC02678 altered the expression of EZH2 downstream targets CDKN1B by regulating EZH2 expression and H3K27me3 mediated by EZH2 (Figure 5E). ChIP-qPCR analysis proved that LINC02678 knockdown decreased the occupancy capacity of EZH2 and H3K27me3 in the promoter regions of CDKN1B and E-cadherin (Figure 5F).

### siEZH2 Reverses LINC02678 Overexpression-Induced Malignancy

**FIGURE 6:**
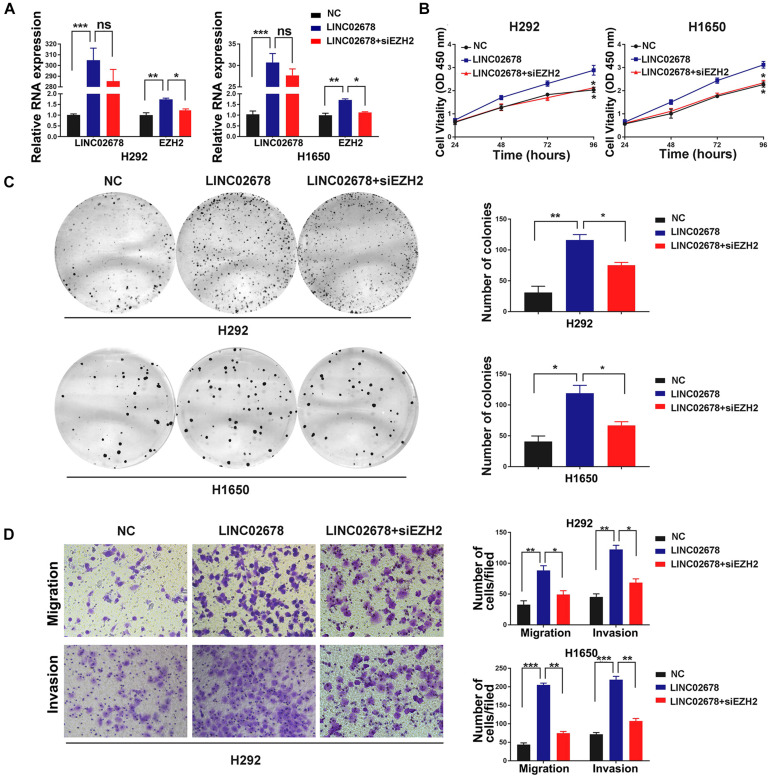
EZH2 is required for LINC02678-induced malignant phenotype. **(A)** RT-qPCR showed LINC02678 and EZH2 expression in H292 and H1650 cells transfected with NC, LINC02678 or LINC02678 + siEZH2. **(B,C)** CCK-8 and colony formation analysis showed the proliferation ability of H292 and H1650 cells after transfected with NC, LINC02678 or LINC02678 + siEZH2. **(D)** Transwell assays were used to estimate metastatic potential in H292 and H1650 cells transfected with NC, LINC02678 or LINC02678 + siEZH2. (*p < 0.05; **p < 0.01; ***p < 0.001. Data were obtained from three independent experiments).

EZH2 has been reported to be associated with the cell cycle, angiogenesis and the capacity of cancer cells to proliferate and migrate (Crea et al., 2012; Yoo and Hennighausen, 2012). To investigate whether EZH2 is required for cancer cell proliferation, migration and invasion that are mediated by LINC02678, NSCLC cells with over-expressed LINC02678 were transiently transfected with siRNA targeting EZH2 (Figure 6A). We observed that siEZH2 partially abrogated the proliferation (Figures 6B,C), migration and invasion (Figure 6D) of NSCLC cells induced by the overexpression of LINC02678.

### LINC02678 Functions as a Potential Oncogenic Factor *in vivo*

**FIGURE 7:**
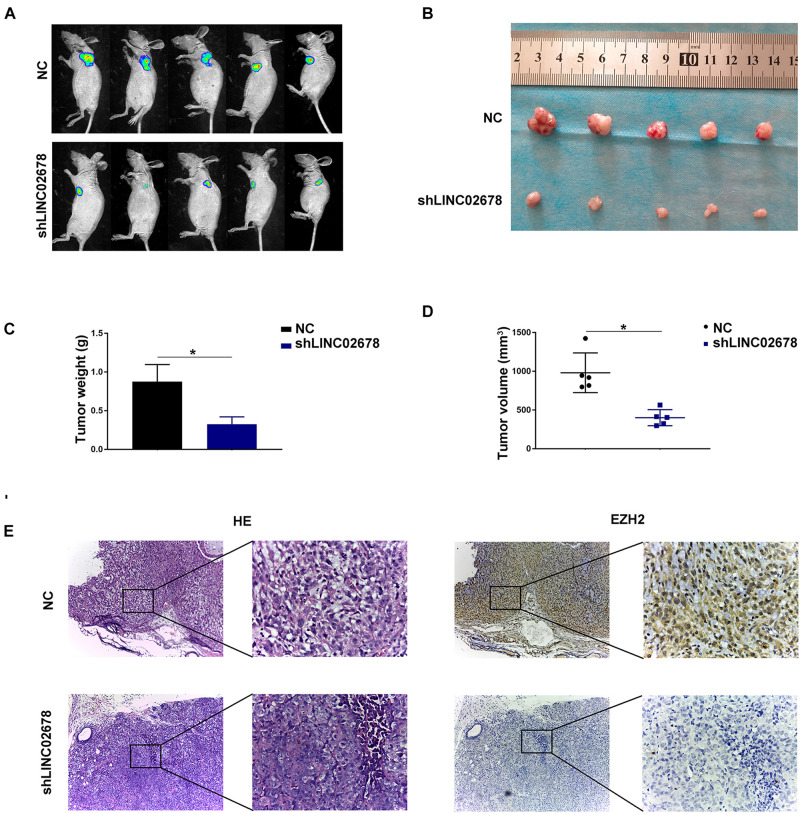
Depletion of LINC02678 repressed tumor growth *in vivo*. **(A)** Representative bioluminescence images of nude mice bearing luciferase-labeled tumors via subcutaneous injection of NC and shLINC02678 groups. **(B)** Representative images of detached tumors from the NC and shLINC02678 groups. **(C)** Histogram of the final tumor weights from NC and shLINC02678 groups. **(D)** Tumor volumes from NC and shLINC02678 groups. **(E)** Representative images of the HE and immunohistochemical staining of EZH2 expression in nude mice xenograft tumor sections (magnification, × 100 and × 400). (*p < 0.05. Data were obtained from three independent experiments).

**FIGURE 8:**
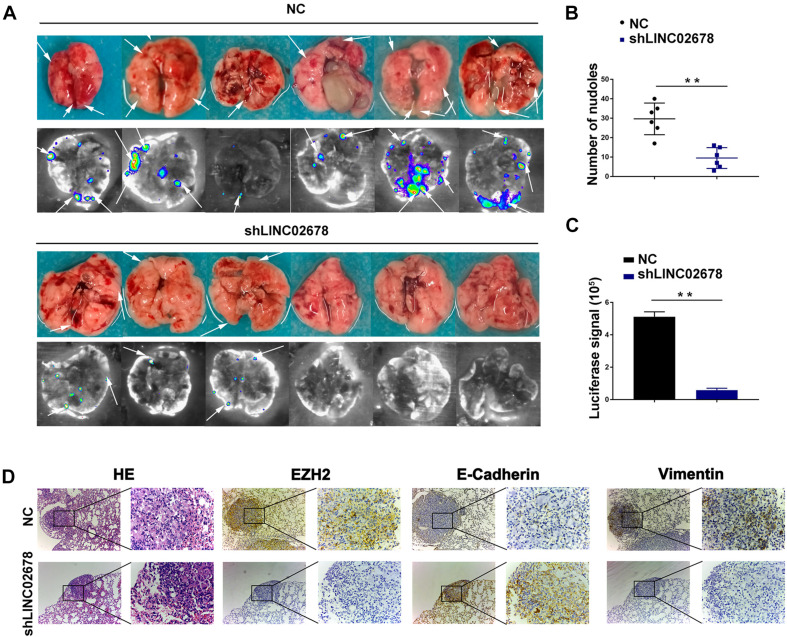
Depletion of LINC02678 repressed tumor metastasis *in vivo*. **(A)** Representative bioluminescence images and lung images were monitored at 60 days after injection with luciferase-labeled cells from NC and shLINC02678 groups. **(B)** Scatter diagram of number of lung metastatic nodules from NC and shLINC02678 groups. **(C)** Histogram depiction of luciferase signal from NC and shLINC02678 groups. **(D)** Representative images of the HE and immunohistochemical staining of E-cadherin, vimentin and EZH2 in nude mice lung metastasis sections (magnification, × 100 and × 400). (**p < 0.01. Data were obtained from three independent experiments).

Mouse transplantation tumor models were used to confirm the function of LINC02678 in tumor growth and metastasis. Aggressive A549 cells were selected to establish cell lines stably transfected with NC and LINC02678 knockdown (shLINC02678) virus for animal experiments. To verify our hypothesis that LINC02678 contributed to tumor growth *in vivo*, NC and shLINC02678 cells were subcutaneously injected into the right dorsal side of the BALB/c mice. Tumor burden in the nude mice was detected at day 28 post-implantation by D-fluorescein-based bioluminescence imaging (Figure 7A). The nude mice injected with shLINC02678 cells exhibited a reduced tumor burden, in comparison with those nude mice injected with NC cells (Figures 7B–E). For the metastasis assay, NC and shLINC02678 cells were injected into the BALB/c mice via the tail vein. The lung metastatic lesions and their bioluminescence images at day 60 after transplantation are shown in Figure 8A. The mice injected with shLINC02678 cells exhibited a significantly lower number of metastatic nodules on lung surface and lower luciferase activity, in comparison with those of the NC group (Figures 8B,C). Furthermore, strong positive immunohistochemical staining for E-cadherin while weak vimentin and EZH2 staining were observed in the shLINC02678 group (Figure 8D). Taken together, our data demonstrated that LINC02678 acts as a promoter of tumor progression *in vivo* by affecting the ability of cancer cells to proliferate and metastasize.

## Discussion

Despite the advances in early diagnosis and treatment for NSCLC, the overall survival rate of the patients with NSCLC remains less than 20% (Siegel et al., 2020). Therefore, it is urgent to explore new markers to guide clinical diagnosis and individualized treatment. In this study, a comprehensive identification and validation were conducted focusing on the upregulated lncRNAs through NSCLC by bioinformatic analysis of genome-wide expression data of LUAD and LUSC in TCGA and GEO. Given that several lncRNAs contribute neutrally or negatively to cancer cells and only a few lncRNAs were identified to regulate tumorigenesis and progression (Garraway and Lander, 2013; Marx, 2014; Pon and Marra, 2015), we conducted a survival analysis based on the screened lncRNAs and the clinical data of NSCLC patients. We found that LINC02678, a lncRNA located on chromosome 10p15.1, was over-expressed and hypomethylated in NSCLC, and it was associated with poor OS and RFS as well as the advanced TNM stages of NSCLC patients.

The sustained proliferation and stimulated invasion and migration are hallmarks of malignancy (Hanahan and Weinberg, 2011). Previous studies have demonstrated that migration, invasion and metastasis are key factors underlying the failure of NSCLC treatment (Wood et al., 2014). Through *in vitro* experiments, we first identified that LINC02678 was highly expressed in NSCLC tissues and regulated cell cycle progression, proliferation and viability of NSCLC cells. E-cadherin, N-cadherin and vimentin are well-recognized markers of EMT, a critical step in metastasis (Pastushenko and Blanpain, 2019). Therefore, we assessed the expression of these three marker proteins in NSCLC cells and confirmed that the overexpressed LINC02678 induced EMT in lung cancer cells, conferring greater aggressiveness to tumor cells. Consistent with these results, *in vivo* experimental animal models also demonstrated the effects of LINC02678 on promoting tumor growth and metastasis.

EZH2 represses gene transcription by catalyzing the trimethylation of lysine 27 of histone H3 (H3K27me3) (Di Croce and Helin, 2013; Grossniklaus and Paro, 2014). EZH2 is associated with the aggressiveness and advanced progression of several types of cancers (Kim and Roberts, 2016). EZH2 also participates in regulating the expression of CDK4/6-cyclin D, CDK2-cyclin E and CDKN1B, which is essential for the cell proliferation (Cao et al., 2012; Geng et al., 2015; Xu et al., 2019). Besides, EZH2 can inhibit the transcription of E-cadherin, thereby inducing EMT (Cao et al., 2008). Our study demonstrates that LINC02678 enhances the inhibitory effect of EZH2 on the downstream proteins CDKN1B and E-cadherin in NSCLC. Besides, EZH2 has been reported to activate signaling pathways associated with the maintenance of lung cancer stem cells, while inhibition of EZH2 could enhance the sensitivity of lung cancer toward chemotherapy (Hussain et al., 2009; Fillmore et al., 2015). However, whether LINC02678 is involved in the regulation of tumor cell stemness and therapeutic resistance through binding potential with EZH2 demands further investigation.

**FIGURE 9:**
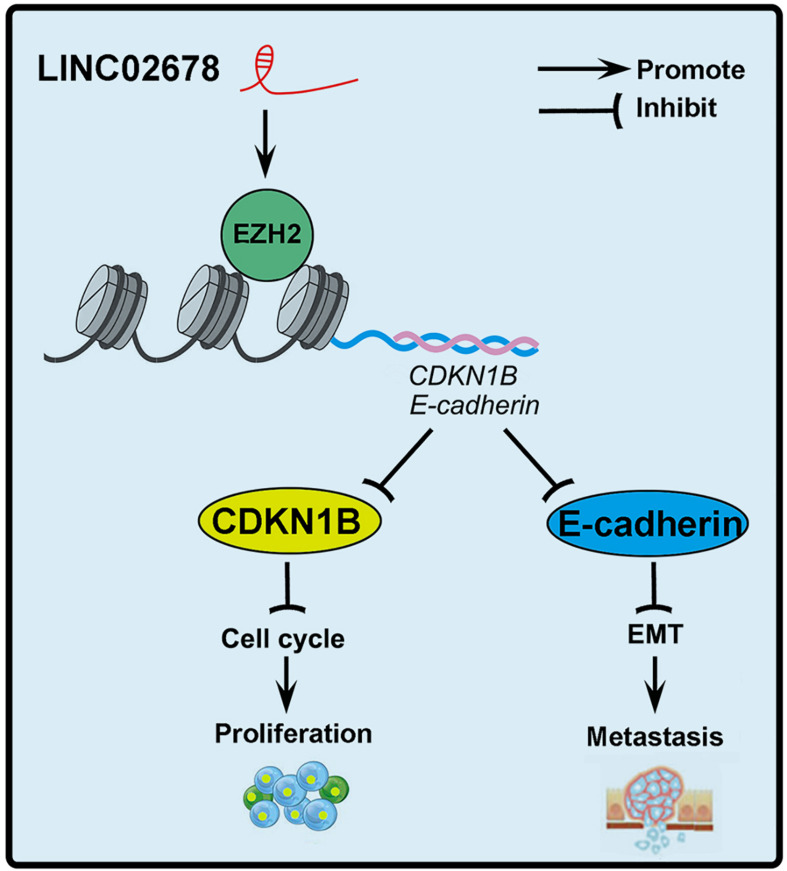
The structural scheme and overall findings of this study. LINC02678 could interact with EZH2 to increase the expression level and activity of EZH2 mRNA and protein. Subsequently, activation of EZH2 epigenetically inhibited the expression of CDKN1B and E-cadherin in NSCLC cells, which promoted cell cycle progression and EMT, and ultimately the proliferation and metastasis of NSCLC cells.

Here, we propose a working model of LINC02678 function in tumor progression in Figure 9. In summary, we identified the lncRNAs that are upregulated in NSCLC. LINC02678, as a novel lncRNA, inhibits the expression of CDKN1B and E-cadherin by binding with EZH2, and ultimately promotes the proliferation and metastasis of NSCLC. Our findings will provide new insights into the diagnostics and individualized treatment of NSCLC.

## Data Availability Statement

The original contributions presented in the study are included in the article/Supplementary Material, further inquiries can be directed to the corresponding author/s.

## Ethics Statement

The studies involving human participants were reviewed and approved by the Ethics Committee of Harbin Medical University. The patients/participants provided their written informed consent to participate in this study. The animal study was reviewed and approved by the Ethics Committee of Harbin Medical University.

## Author Contributions

LC, XL, YX, and DJ designed this research. YZ and DJ carried out most of the experiments, analyzed the data, drew the figures, and drafted this manuscript. MC, FT, WF, JH, and DJ helped with cell culture, western blotting experiments, RT-qPCR, CHIP, RIP and the CCK-8 assay. YCi, RG, and YCa provided the technical support for bioinformatics analysis. SZ and YL helped with the animal experiment. YX and YZ helped check the manuscript and figures. All authors read and approved the final manuscript.

## Conflict of Interest

The authors declare that the research was conducted in the absence of any commercial or financial relationships that could be construed as a potential conflict of interest.

## Footnotes

1 http://www.ncbi.nlm.nih.gov/geo/

2 https://cancergenome.nih.gov/

3 http://lilab.research.bcm.EdU/cpat/

4 http://cpc.cbi.pku.EdU.cn/
