# Supplementary material for: LINC02678 as a Novel Prognostic Marker Promotes Aggressive Non-small-cell Lung Cancer
Source: Front Cell Dev Biol. 2021 May 28;9:686975. doi: 10.3389/fcell.2021.686975 (PMC8194704; doi:10.3389/fcell.2021.686975)
Supplement: Supplementary file 1 [file Data_Sheet_1.docx]

Supplementary Material

## Supplementary Figure legends

**Supplementary Figure 1.** The promoter methylation levels of LINC02678. **(**A**)** The promoter methylation levels of LINC02678 in human LUAD, LUSC tissues and normal lung samples were analyzed with TCGA datasets.

**Supplementary Figure 2.** shLINC02678 inhibits proliferation and G1/S transition of NSCLC cells. **(A)** RT-qPCR analysis showed the expression of LINC02678 in 95D and A549 cells transfected with a negative control shRNA (NC) or two shRNAs targeting LINC02678 (shLINC02678-1 and shLINC02678-2). **(B)** CCK-8 analysis showed the proliferative ability of 95D and A549 cells in NC and shRNAs (shLINC02678-1 and shLINC02678-2) groups. **(C-D)** Colony formation and EdU analysis show the proliferative ability of 95D and A549 cells in NC and shRNAs groups. **(E)** Flow cytometry analysis showed the proportion of 95D and A549 cells in G0/G1, S and G2/M phases in NC and shRNAs groups. **(F)** Western blotting analysis showed the expression of key cell cycle-related protein (CDK4, CDK6 and CyclinD1) in NC and shRNAs groups. (**p*<0.05; ***p*<0.01; ****p*<0.001. Data were obtained from three independent experiments).

**Supplementary Figure 3.** The suppressive role of shLINC02678 in cell migration, invasion and EMT of NSCLC cells. **(A-B)** The effects of LINC02678 knockdown on the migration and invasion of 95D and A549 cells were evaluated by wound healing experiment and Transwell assay. **(C)** Evaluation of EMT-related markers performed by RT-qPCR and western blotting in 95D and A549 cells with knockdown of LINC02678. (**p*<0.05; ***p*<0.01; ****p*<0.001. Data were obtained from three independent experiments).
