# Supplementary figures and images for: LINC02678 as a Novel Prognostic Marker Promotes Aggressive Non-small-cell Lung Cancer
Source: Front Cell Dev Biol. 2021 May 28;9:686975. doi: 10.3389/fcell.2021.686975 (PMC8194704; doi:10.3389/fcell.2021.686975)

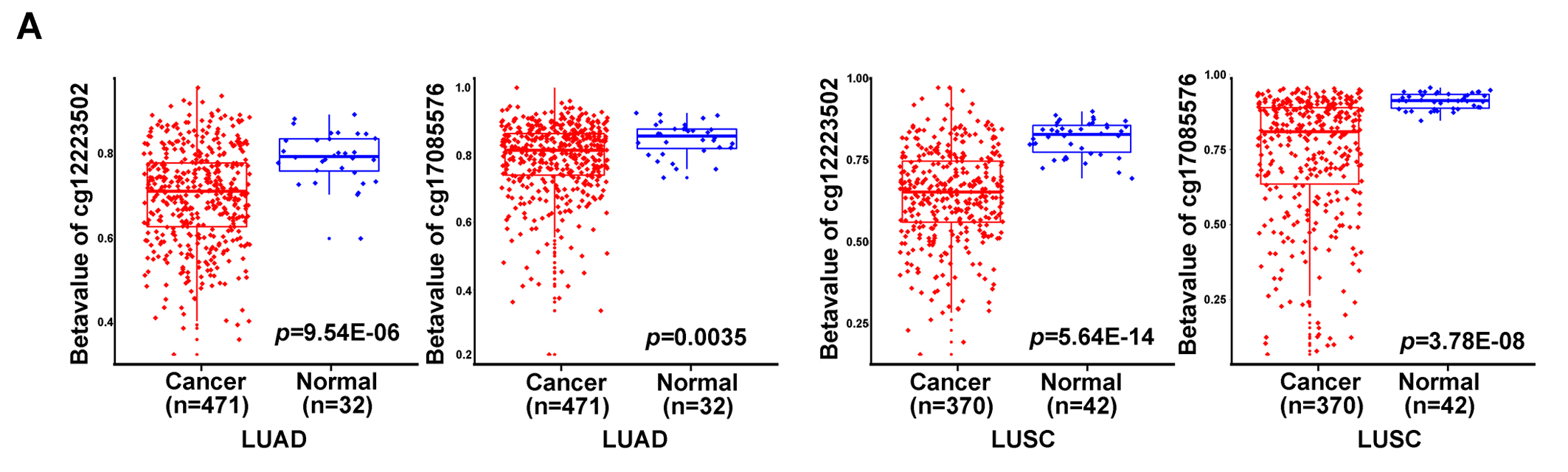

Supplement: Supplementary file 2 [file Image_1.JPEG]

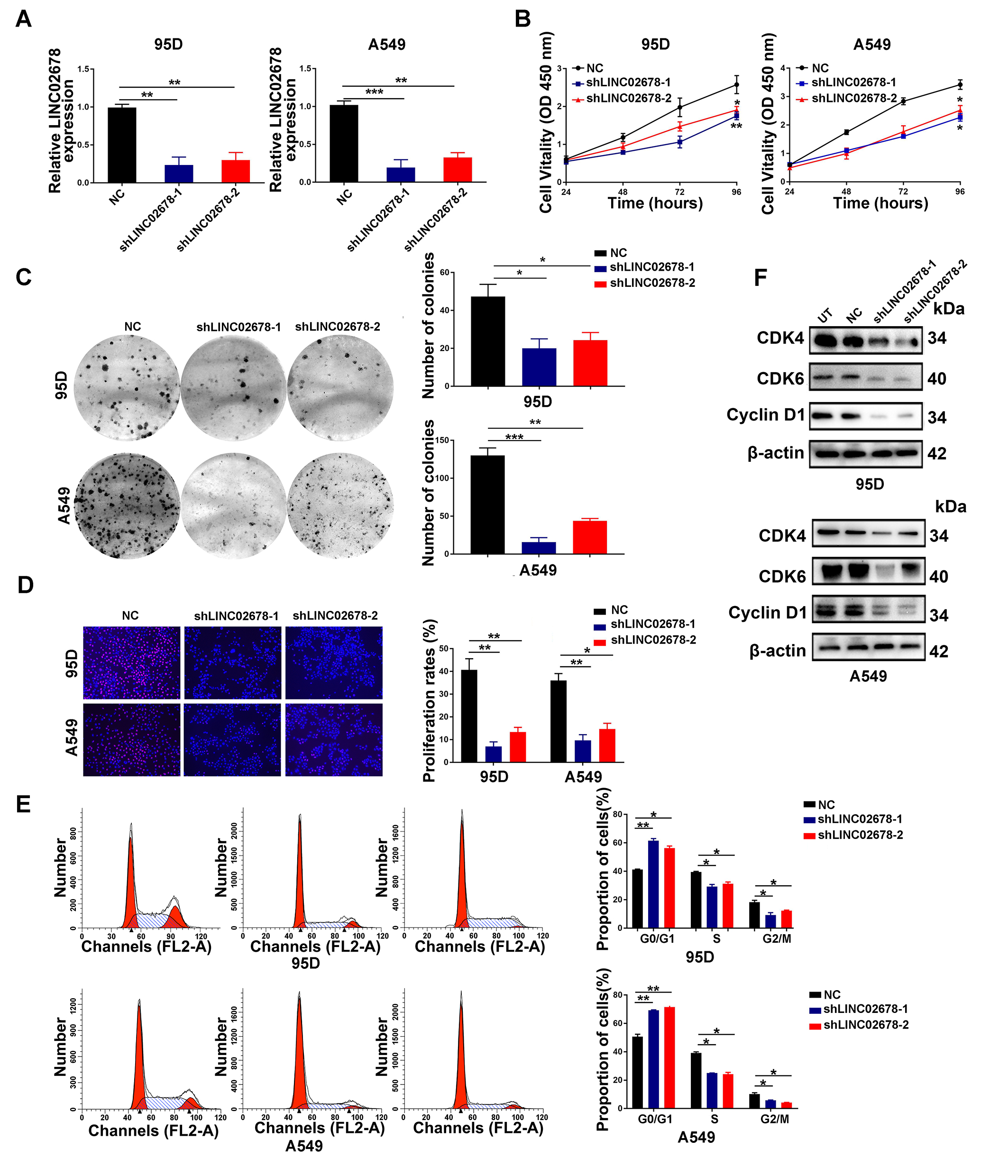

Supplement: Supplementary file 3 [file Image_2.JPEG]

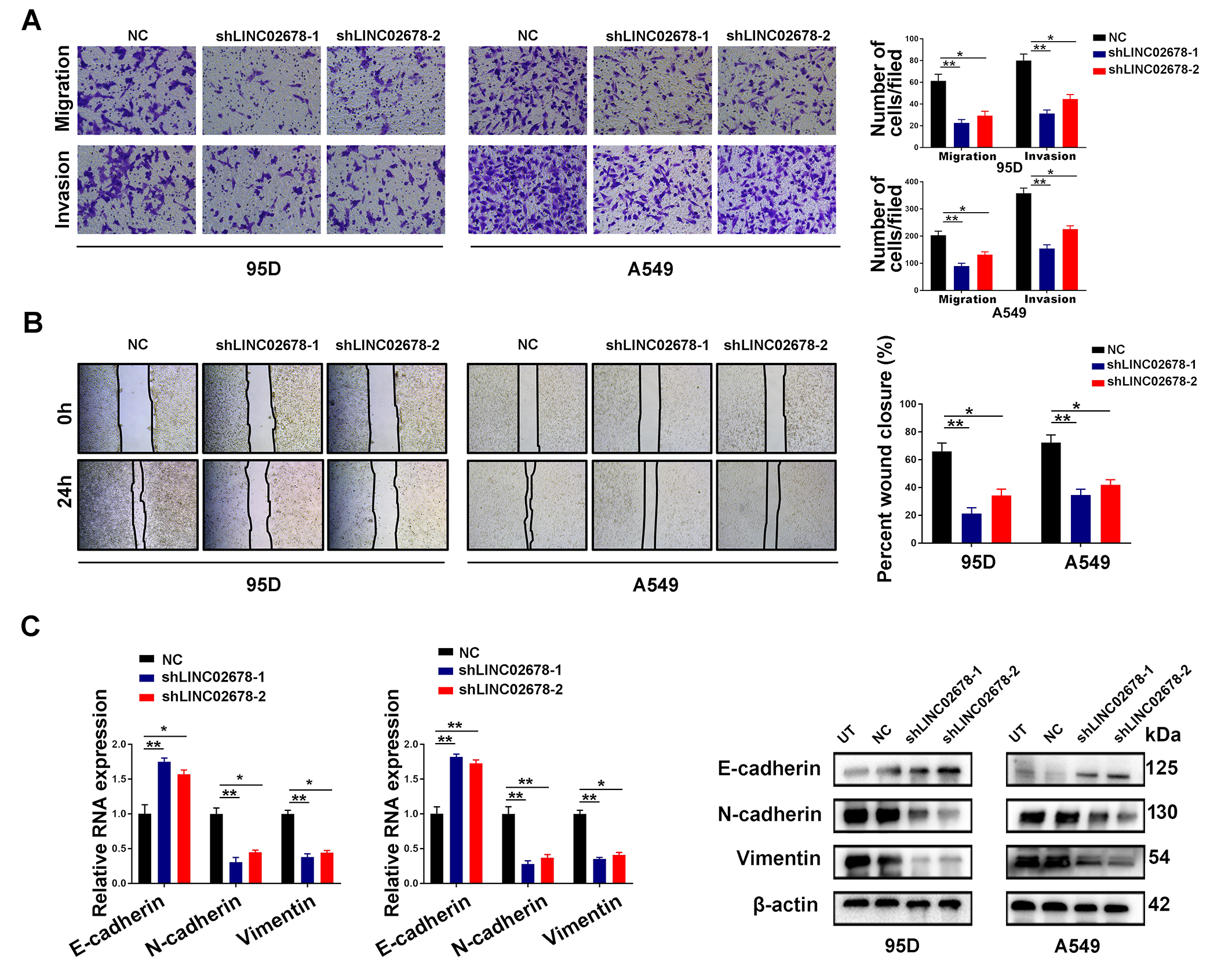

Supplement: Supplementary file 4 [file Image_3.JPEG]
